# Characterizing left ventricular systolic dysfunction in sickle cell disease: three contrasting case reports

**DOI:** 10.1093/ehjcr/ytae383

**Published:** 2024-08-14

**Authors:** George Katis, Sridhar Srinivasan, Perla Eleftheriou, Malcolm Walker, Polyvios Demetriades

**Affiliations:** University College Hospital, 235 Euston Road, London NW12BU, UK; University College Hospital, 235 Euston Road, London NW12BU, UK; University College Hospital, 235 Euston Road, London NW12BU, UK; University College Hospital, 235 Euston Road, London NW12BU, UK; University College Hospital, 235 Euston Road, London NW12BU, UK

**Keywords:** Case report, Sickle cell disease, Heart failure, Cardiac MRI, Left ventricular systolic dysfunction

## Abstract

**Background:**

Left ventricular systolic dysfunction (LVSD) is an uncommon but life-threatening complication of sickle cell disease (SCD), with poorly characterized aetiology. We present three SCD patients with LVSD due to different underlying mechanisms.

**Case summary:**

The first case describes rapid deterioration in LV function secondary to severe cardiac iron overload in a 37-year-old female with poor chelation compliance after 10 years of top-up transfusions for SCD. The second case is a severe non-ischaemic dilated cardiomyopathy (DCM) in a 42-year-old SCD patient with longstanding sickle nephropathy and hypertension. The final case demonstrates severe LVSD with large transmural infarcts (ischaemic DCM) in the absence of epicardial coronary disease in a 52-year-old SCD patient.

**Discussion:**

This case series presents the first attempt to characterize the aetiology of LVSD in SCD. We identified three phenotypes: iron-overload cardiomyopathy, non-ischaemic DCM, and ischaemic DCM. These contrasting cases highlight the significance of understanding the underlying pathology in determining individualized treatment plans for these high-risk patients. We discuss the role of cardiac MRI (CMR) in characterizing LV dysfunction, and we believe that this case series will form the basis of prospective studies to further delineate the pathophysiology of LVSD in SCD.

**Learning points**

- LV systolic dysfunction in sickle cell disease (SCD) is rare but can have devastating consequences.
- Characterizing the underlying aetiology allows individualized treatment plans.
- Comprehensive cardiac MRI (CMR) studies with late gadolinium enhancement and parametric mapping are recommended in characterizing the aetiology of LVSD in SCD.
- Joined cardiology–haematology MDT approach is helpful in challenging cases.

## Introduction

Sickle cell disease (SCD) is a monogenetic disorder due to a point mutation in the haemoglobin β-globin gene that results in the production of HbS (sickle haemoglobin). This leads to vaso-occlusion resulting in organ ischaemia and tissue necrosis as well as chronic haemolytic anaemia.^1^ Patients often require regular top up or red cell exchange transfusions (RCE) to prevent complications. Cardiac complications include LV diastolic dysfunction (LVDD) and pulmonary hypertension.^1^ LV systolic dysfunction (LVSD) is an uncommon, poorly characterized, but life-threatening complication. We describe three SCD cases who were found to have LVSD due to different underlying mechanisms.

## Summary figure

**Table:** 

	Case 1	Case 2	Case 3
ECHO findings	Normal LV function (6 weeks before CMR)	Severe LVSD	Severe LVSD
Cardiac MRI (CMR) findings	Normal LV size with severe LVSD, severe cardiac iron loading	Dilated LV with moderate LVSD, no cardiac iron, and non-ischaemic LGE, high T1 values	Severe LVSD with two large transmural infarcts, no cardiac iron
Diagnosis	Iron-overload related cardiomyopathy in a sickle cell patient on regular top-up transfusions	Non-ischaemic dilated cardiomyopathy in SCD in the context of HTN and ESRF	Ischaemic cardiomyopathy secondary to microvascular disease in SCD
Management	Deferoxamine infusions, heart failure medications commenced	Heart failure medication optimization	Heart failure medication optimization
Outcomes	Repeat CMR showed improvement in LV function and marginal reduction in cardiac iron.	Repeat TTE showed persistently severe LVSD. For consideration for ICD	Recurrent heart failure admissions. Declined for advanced HF therapies. Palliated and passed away

## Case presentation

### Case 1

**Figure 1:**
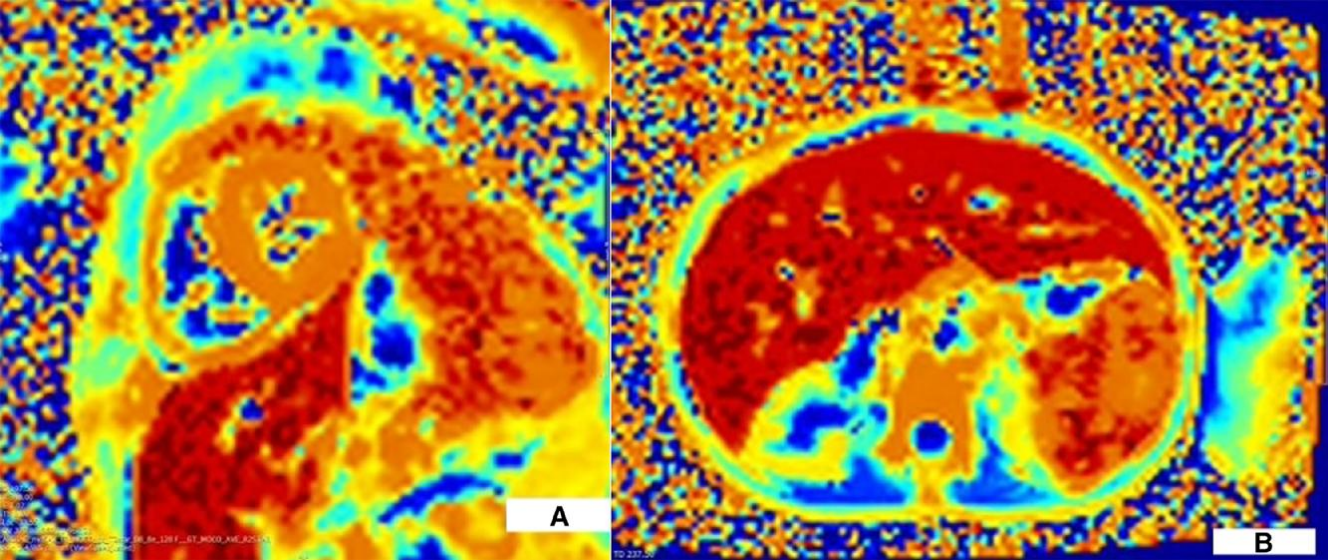
Case 1 T2* sequences showed severe iron loading in the heart with T2* 7 ms (*A*) and liver with T2* 1.2 ms (*B*).

A 37-year-old female with SCD HbSS, frequent vaso-occlusive crises, a 10-year history of regular top-up transfusions, and poor iron chelation compliance (deferasirox 1080 mg o.d. and deferiprone 1500 mg o.d.). She presented acutely with peripheral oedema and breathlessness, requiring inpatient admission. Serum ferritin was significantly elevated at 11 106 μg/L [normal range 13–150 μg/L]. Transthoracic echocardiography (TTE) revealed normal left ventricular systolic function with intermediate probability of pulmonary hypertension, moderate tricuspid regurgitation (TR), and normal diastolic function (LVEF 60–65%, LVIDD 4.45 cm, LVIDS 3.05 cm, PASP 43 mmHg, TR jet velocity 3.2 m/s). She was treated with intravenous diuretic therapy (furosemide 40 mg o.d.) in view of elevated NTproBNP 717 ng/L (<50), possibly reflecting a degree of diastolic dysfunction due to iron overload, despite normal diastolic parameters on TTE. Urgent outpatient cardiac MRI (CMR) 4 weeks later revealed severe biventricular failure with normal biventricular size (LVEF 31%, LVEDV 94 mL, LVESV 65 mL) (see Supplementary material online, *Videos S1* and *S4*), severe cardiac (T2* 7 ms, normal range > 20 ms) and severe liver (T2* 1.2 ms, normal range > 17 ms) iron loading (*Figure 1*). Following a cardiology–haematology multi-disciplinary team meeting, she was commenced on 12-hourly desferrioxamine infusion and converted to RCE. Heart failure medications were optimized, with doses limited by poor tolerance (ramipril 1.25 mg o.d., bisoprolol 1.25 mg o.d.). Repeat CMR, 10 weeks later, showed significant improvement in LV systolic function, however persistent significant cardiac iron loading (T2* 5.1 ms) (see Supplementary material online, *Videos S5* and *S6*). She remains clinically well to date.

### Case 2

**Figure 2:**
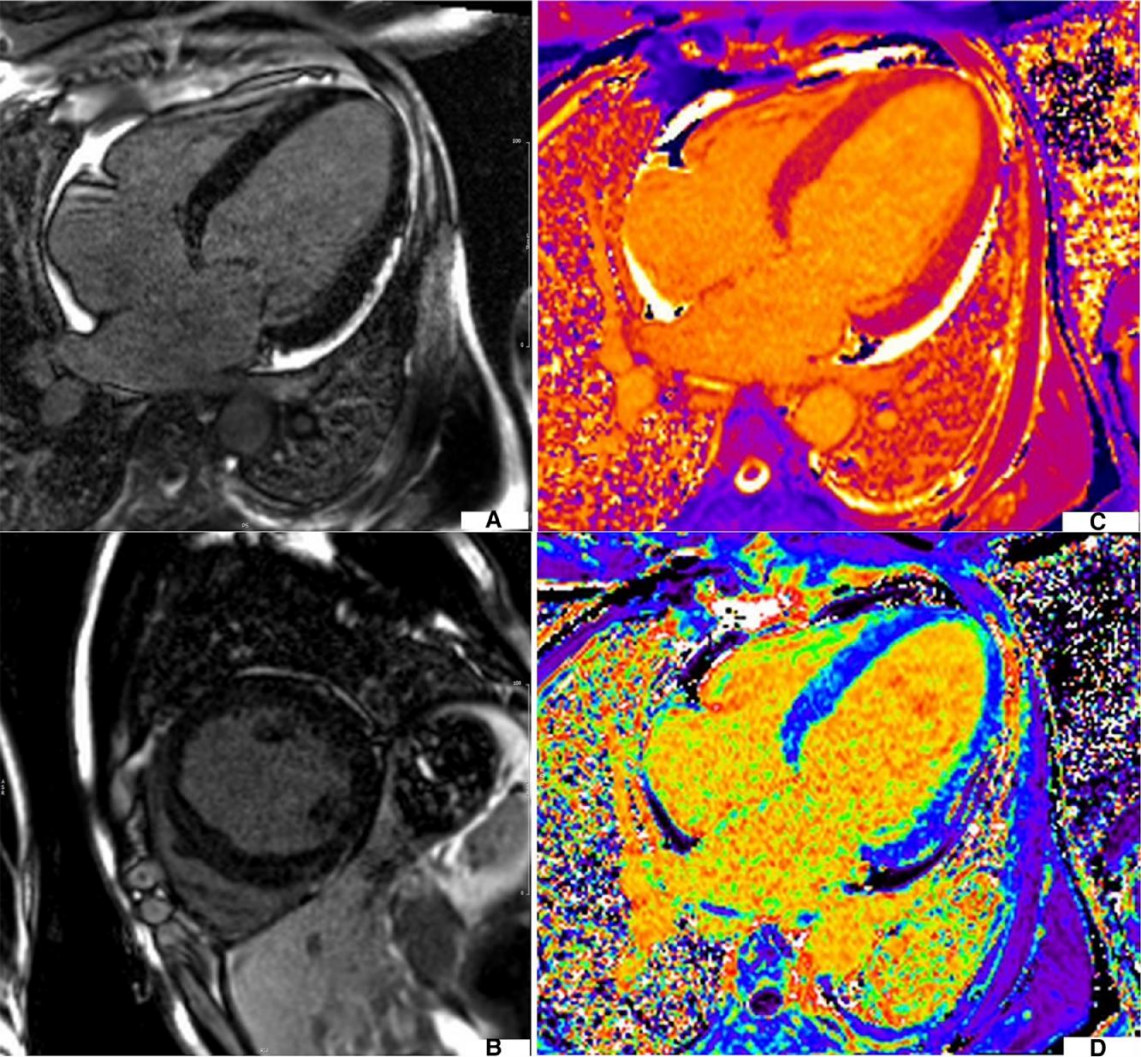
Case 2 LGE imaging showing mid wall late enhancement in the septum and patchy late enhancement elsewhere (*A*, *B*). T1 and ECV values were elevated (*C*, *D*).

A 42-year-old male with HbSS genotype SCD, on monthly RCE, and end-stage renal failure (ESRF) secondary to sickle nephropathy. Surveillance TTE showed severe LV dilatation and severe LVSD with intermediate probability of pulmonary hypertension (LVEF 35%, LIVDD 6.84 cm, LVIDS 5.57 cm, TR Vmax 3.3 m/s, PASP 51 mmHg, E/e′ 25). Previous TTE a year prior had shown dilated LV with preserved function. Contrast-CMR (*Figure 2*) with parametric mapping confirmed dilated LV, eccentric remodelling, and moderate global LVSD (LVEF 39%, LVEDV 345 mL, LVESV 210 mL, ECV 32%) (see Supplementary material online, *Video S2*). There was diffuse elevation of T1 (1188 ms, normal range 970–1050 ms on 1.5 T), with associated non-ischaemic late gadolinium enhancement (LGE) in the septum. There was no evidence of myocardial iron loading (T2* 45 ms, normal range > 20 ms) or previous myocardial infarction. Findings were in keeping with non-ischaemic dilated cardiomyopathy (DCM). Heart failure medications were optimized in conjunction with the renal team’s guidance (irbesartan 75 mg o.d., bisoprolol 10 mg o.d.). Since then, he has had recurrent admissions with decompensated heart failure and latest TTE showed persistently severe LVSD. He is currently being considered for primary prevention implantable cardiac defibrillator (ICD).

### Case 3

**Figure 3:**
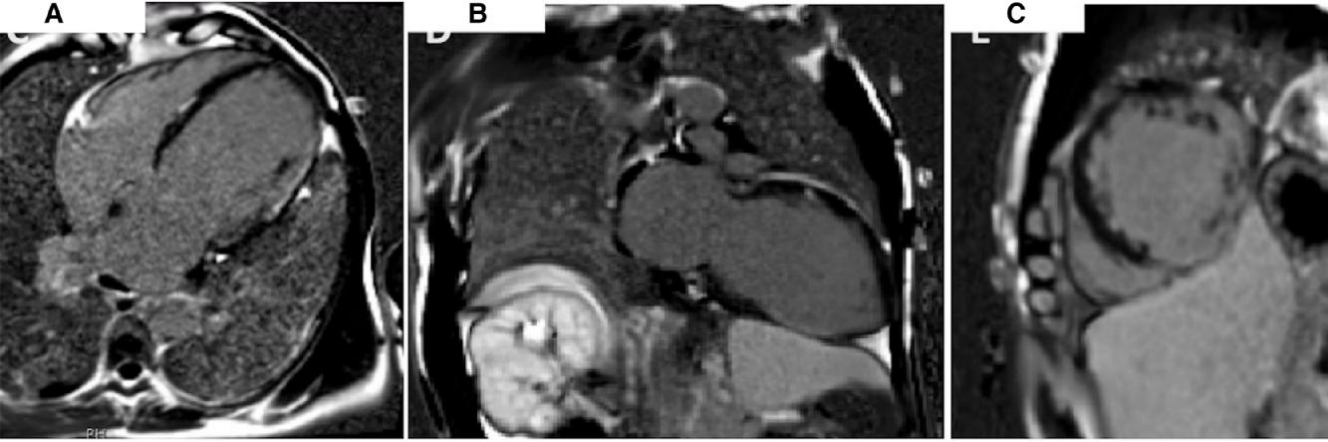
Case 3 LGE shows extensive transmural infarcts in the inferior and lateral walls (*A–C*).

A 52-year-old male with SCD HbSS, previous stroke, currently not on disease modifying treatment. He presented with NYHA III breathlessness, peripheral oedema and ascites to his local hospital, and was found to have severe LVSD on TTE (LVEF 25%, LVIDD 6.93 cm, LVIDS 6.16 cm, TR Vmax 2.21, E/e′ 7.19). CMR showed severely dilated LV, severe LVSD (LVEF 32%, LVEDV 518 mL, LVESV 351 mL, ECV 30%, T2* not reported), and multiple regional wall motion abnormalities (see Supplementary material online, *Video S3*). Late gadolinium enhancement imaging confirmed large transmural infarcts in lateral and inferior walls (*Figure 3*). CT coronary angiogram excluded epicardial coronary disease, showing no visible plaque. Despite further optimization of heart failure therapy (ramipril 1.25 mg o.d.; bisoprolol 1.25 mg o.d., dapagliflozin 10 mg o.d., spironolactone 25 mg o.d.), there was no clinical or symptomatic improvement. He was deemed not suitable for advanced heart failure therapy and continued to deteriorate before passing away in hospital.

## Discussion

Common cardiac complications in SCD include LVDD that is a result of chronic anaemia and haemolysis.^1^ Chronic tissue hypoxia results in an increase in cardiac output and significant dilation of the LV, leading to an increased stroke volume.^1^ The dilated ventricle develops eccentric hypertrophy that initially maintains sufficient cardiac output, however over time the increased wall stress leads to increasing LV mass and impaired LV filling,^2^ as well as left atrial dilatation. Diastolic dysfunction has also been shown to contribute to pulmonary hypertension, a combination that gives an extremely poor prognosis in SCD.^3^

The mechanism behind LVSD in SCD is scarcely described in literature and remains poorly characterized. Our cases demonstrated three different phenotypes of LVSD in SCD: iron-overload cardiomyopathy, non-ischaemic DCM, and ischaemic DCM.

Case 1 highlights that iron overload can be a cause of LVSD in SCD. In contrast to other haemoglobinopathies that require transfusions, it is rare for SCD patients to develop cardiac iron loading—Tavares *et al.*^4^ report a prevalence of <3% in their cohort. There are multiple explanations for this. Firstly, the majority of haemolysis in thalassaemia occurs in the bone marrow whereas it is predominantly intravascular in SCD, resulting in high turnover of new cells and excretion of urinary and biliary iron.^5^ In addition, the chronic inflammatory state caused by recurrent vaso-occlusion means that iron becomes trapped in tissues rather than blood.^4^ Finally, SCD patients often undertake RCE rather than top-up transfusions, which contribute to less iron overload.^4^ Despite the rarity of iron overload in SCD, case 1 demonstrates that it can result in rapid deterioration in LV function with devastating results if not identified and treated promptly.

Non-ischaemic DCM in SCD has been previously linked to ESRF and hypertension such as in case 2.^1^ CMR identified significantly elevated T1 and non-ischaemic LGE suggestive of fibrosis. Desai *et al*.^6^ confirmed histologic fibrosis in 25% of their cohort with SCD, describing three patterns at autopsy that correlate to LGE seen on CMR. We suspect that the ‘non-ischaemic DCM’ phenotype of LVSD in SCD may represent a late consequence of the chronic pathophysiologic adaptations described in SCD in the context of diastolic dysfunction secondary to anaemia, chronic renal failure and hypertension.

Finally, case 3 demonstrates the ‘ischaemic DCM’ phenotype. As in our case, literature suggests that myocardial infarcts in SCD are often secondary to microvascular disease as epicardial coronary disease is exceedingly rare.^6,7^ Quantitative perfusion using CMR may prove helpful in the future in further characterizing microvascular disease in SCD.

To our knowledge, these three cases present the first attempt to characterize the aetiology of LVSD in SCD. Understanding of the underlying pathophysiological cause will allow individualized changes in patients’ treatments, such as addressing iron overload or adapting heart failure medication. Conventional heart failure therapies maybe helpful although there is no evidence of effectiveness in this clinical context. We emphasize the vital role of CMR in phenotyping LVSD in SCD, and we believe that this short case series will support the undertaking of prospective studies to further delineate the underlying pathophysiology of these very serious complications of SCD.

## Lead author biography


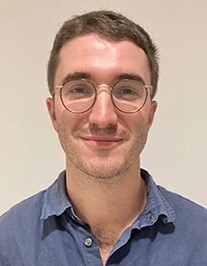


George Katis (BMedSci, BMBS) is a graduate of University of Nottingham and is currently completing Internal Medical Training (IMT) in London, UK. He worked abroad for 2 years in Auckland, New Zealand before returning to the UK for training. His interest is in cardiology, and he is looking to apply for specialty training after completing IMT.

## Supplementary Material

ytae383_Supplementary_Data

## Data Availability

The data underlying this article will be shared on reasonable request to the corresponding author.

## Supplementary material

**Consent:** The authors confirm that written consent for submission and publication of this case report including images and associated text has been obtained in line with COPE guidelines from the patient.

**Funding:** Not applicable.
